# Characterization and Comparison of Nutritional Intake between Preparatory and Competitive Phase of Highly Trained Athletes

**DOI:** 10.3390/medicina54030041

**Published:** 2018-05-30

**Authors:** Catarina L. Nunes, Catarina N. Matias, Diana A. Santos, José P. Morgado, Cristina P. Monteiro, Mónica Sousa, Cláudia S. Minderico, Paulo M. Rocha, Marie-Pierre St-Onge, Luís B. Sardinha, Analiza M. Silva

**Affiliations:** 1 Faculdade de Ciências da Nutrição e Alimentação da Universidade do Porto, Rua Dr. Roberto Frias, 4200-465 Porto, Portugal; catnunes94@gmail.com; 2 Exercise and Health Laboratory, CIPER, Faculdade de Motricidade Humana da Universidade de Lisboa, Estrada da Costa, 1499-688 Cruz-Quebrada, Portugal; CMatias@fmh.ulisboa.pt (C.N.M.); dianasantos@fmh.ulisboa.pt (D.A.S.); morgado.ze@gmail.com (J.P.M.); cmonteiro@fmh.ulisboa.pt (C.P.M.); cminderico@gmail.com (C.S.M.); procha@fmh.ulisboa.pt (P.M.R.); lsardinha@fmh.utl.pt (L.B.S.); 3 Nutrition & Metabolism, NOVA Medical School|Faculdade de Ciências Médicas, Universidade NOVA de Lisboa, Lisboa, 1169-056, Portugal; monica.sousa@nms.unl.pt; 4 CINTESIS-Center for Health Technology and Services Research, R. Dr. Plácido da Costa, 4200-450, Porto, Portugal; 5 New York Obesity Nutrition Research Center and Institute of Human Nutrition, Columbia University Medical Center, New York, NY 10032, USA; ms2554@columbia.edu

**Keywords:** diet composition, fluids, sports, body composition, macronutrients

## Abstract

*Background and objective:* For a high level athlete, it is essential to ensure optimal energy as well as macro- and micro-nutrient and fluid intakes, in order to improve their performance during training and competition. Protein intake should be 1.2–2.1 g/kg/d, whereas the requirements for carbohydrate and fat intakes should be >5g/kg/d and 20–35% of energy, respectively. The micronutrient and fluid intakes in athletes were compared to the Dietary Reference Intake (DRI) and European Food Safety Authority (EFSA) recommendations, respectively. This study aimed to characterize and compare the nutritional habits of athletes at the preparatory and competitive phase, and to test if their nutritional intakes were in accordance with the recommendations. *Materials and methods:* A total of 276 professional athletes were assessed. To evaluate their nutritional intake, the athletes completed a 7 days food record. Under reporting was defined using a ratio of energy intake to basal metabolic rate (BMR) of 1.1. Body composition was assessed using dual energy X-ray absorptiometry (DXA). *Results:* Almost half (49%) of the athletes from the final sample reported lower measured intakes of carbohydrates and 27% reported a higher consumption of proteins than what was recommended. In both the preparatory and competitive phases, the micronutrients with a higher mismatch between the actual and recommended intakes were vitamins D and E, magnesium, folate, calcium, and zinc for both sexes, and iron intake for females. A large proportion of athletes reported a lower water intake. Compared to the recommendations, males reported a higher intake of carbohydrates, lipids, vitamins E, calcium, and magnesium (*p* <0.05) in the competitive phase, while females reported a lower ingestion of water, vitamins A and D, and calcium (*p* <0.05) in the preparatory phase. *Conclusions:* Overall, in the preparatory and competitive phases of the season, athletes reported a macro- and micro-nutrient intake below the recommendations, especially in the female athletic population. Dietary intakes in athletes need to be optimized and adjusted to their requirements, according to sex and sport, so as to avoid compromising health and performance.

## 1. Introduction

An optimal intake of energy, macro- and micro-nutrients, and fluids are essential for good performance in all sports [1]. According to the sports guidelines, protein intakes should be 1.2–2.1 g/kg/d [2], but higher values are seen in strength/power training athletes and bodybuilders [3]. For carbohydrates, Burke et al. considered intakes of at least 5 g/kg/d (for a moderate exercise program, such as 1 h/d) for fuel and recovery [4]. Fat intakes should be between 20–35% of the energy intake. An adequate energy intake is important for optimal protein use and performance [5]. In 2014, Desbrow et al. showed that the guidelines for the adult athlete could be considered for adolescent athletes [6].

Dehydration decreases exercise performance, which results in an increased core temperature, heart rate, perception of effort, and cortisol [7]. Water requirements vary between individuals and are affected by daily training activity and environmental conditions. High cortisol levels increase the risk of infectious illness if hard training is performed consistently in an inadequate hydration status [7]. Therefore, it is important to ensure an adequate daily fluid intake, especially before, during, and after exercise, in order to achieve an optimal performance [5]. The European Food Safety Authority (EFSA) has considered that the Adequate Intake (AI) for water for individuals between 9–13 years of age is 2.1 L for boys and 1.9 L for girls. For those ≥14 years of age, the recommendations are 2.5 L for males and 2.0 L for females [8].

In addition, adequate micronutrient intakes can enhance recovery and improve sports performance [9]. Athletes should at least meet the Dietary Reference Intake (DRI) for micronutrients, because of the wide safety margins for nutrient recommendations [5], and should pay special attention to the optimal intake of iron, vitamin D, calcium, and antioxidants [10]. The requirements of micronutrients, in particular sodium, B6, and iron, may be dependent on the levels of physical activity [8], but further research is needed. Besides this, the DRI for micronutrients seems to be appropriate for most athletes [2], except for iron, which is 1.3–1.7 times higher for athletes [11]. Athletes have a higher energy expenditure, which, in theory, leads to an increase of food intake. This higher consumption may lead to a higher intake of micronutrients, especially if the athlete has healthy food choices [10]. However, this does not seem to happen as a result of an unbalanced diet (low density diet), which leads to a lower micronutrient intake than expected [12].

For those who restrict energy intakes or specific food groups so as to achieve weight loss, more attention to micronutrient adequacy is required, because of the higher risk of deficiency.

Despite all of the attention that is given by the scientific community to the nutritional recommendations for athletes [2,4], some recent studies have reported that athletes still do not achieve the recommended intakes of energy, and macro- and micro-nutrients. Several studies have shown that carbohydrate intakes can be below the recommended amount [13,14,15] and, sometimes, protein intakes can be higher [13,15]. Athletes, particularly females, also reported an inadequate intake of vitamin E, calcium, folate, and magnesium [15,16].

Wardenaar et al. (2017) investigated 553 Dutch elite and sub-elite athletes to understand whether they met the micro-nutrient recommendations and whether the adequacy of their intake was related to the use of dietary supplements, sport nutrition products, or a combination, using a 24 h dietary recall plus a nutritional supplement questionnaire. The Dutch athletes reported a lower intake of micronutrients, such as iron; vitamin D; vitamin B1 and B2; vitamin A, B3, and C; and selenium [10]. Recently, Marius et al. [15] reported inadequate carbohydrate intakes in 80% of Lithuanian athletes. Similar findings have been reported by others [14,16,17,18,19,20,21].

Previous studies have various limitations, including small sample sizes [16,19] and that the time at which the data are obtained is frequently not reported. This is relevant because the nutritional needs of athletes varies, depending on training intensity, which is much different in the different phases of rest, training, and competition. Few studies have compared the nutritional intakes between the season phases. Knowing athletes’ nutritional habits and their variation throughout the sport season is important so as to provide a better follow-up and guidance during the season. Addditionally, this can help to develop strategies to achieve dietary recommendations in each phase of the sport season [2]. The aim of the present study was two-fold, namely: (1) to characterize and compare the dietary habits and nutritional intake of male and female athletes in two phases, preparatory and competition, and (2) to compare the nutritional intakes with the recommendations.

## 2. Methods

### 2.1. Participants

This study enrolled 276 professional athletes that participated in several sports, namely: basketball, judo, rowing, handball, volleyball, swimming, karate, triathlon, tennis, athletics, sailing, shooting, fencing, and horseball. These athletes were evaluated at the beginning of the preparatory phase. Of those, 85 were also evaluated in the competitive phase. Informed consent was obtained from all of the athletes and, for those under the age of 18 years, the participants’ tutors were informed about the possible risks of the investigation before giving written informed consent to participate. All of the procedures were approved by the Ethics Committee of the Faculty of Human Kinetics, University of Lisbon (Lisbon, Portugal) and were conducted in accordance to the declaration of Helsinki, for human studies from the World Medical Association [22]. The participant inclusion criteria were as follows: (a) training >10 h/week; (b) two negative test outcomes for performance enhancing drugs; and (c) not taking any medications.

Medical screening indicated that all of the participants were in good health, and were without endocrine abnormalities that would have limited their participation in the study.

### 2.2. Experimental Design

All 276 athletes were evaluated at the beginning of the preparatory phase. Of those, 85 athletes participated in an observation study with a follow-up over one season (evaluated at the preparatory as well as during the main stage of the competition) ranging from 5 to 10 months, depending on each sport’s competitive timetable (~5 months for volleyball, ~7 months for handball, ~8 months for basketball, ~7 months for swimmers, and ~10 months for triathletes). For these sports, the first moment of evaluation corresponded to the beginning of the season, while the second period was the competition phase, which started just before the main stage of the competition. During the competitive phase, competitions were relegated to weekends (at least one game per weekend) and were integrated into the National Championships. For the remaining sports, an observational cross-sectional study was used to assess those athletes in the preparatory phase of the season.

### 2.3. Body Composition Measurements

Body composition assessments during both of the measurement periods were carried out in the morning following a 12 h fast, with no vigorous exercise within 15 h, and no caffeine and alcohol intake during the preceding 24 h. All of the measurements were performed by the same technician.

### 2.4. Anthropometric Measurements

Participants were weighed to the nearest 0.1 kg and their height was measured to the nearest 0.1 cm with a stadiometer (Seca, Hamburg, Germany), according to standardized procedures that were described elsewhere [23]. Body mass index (BMI) was calculated as the body mass (kg) divided by the square of stature (m). The measures were taken twice, using the average as the final value [23].

### 2.5. Dual Energy X-ray Absorptiometry (DXA)

The athletes underwent a whole-body DXA scan, according to the procedures that were recommended by the manufacturer, on a Hologic Explorer-W fan-beam densitometer (Hologic, Waltham, MA, USA). Scan positioning, acquisition, and analysis were standardized. The whole-body measurements of the bone mineral content (BMC), absolute fat mass (FM), percent FM (%FM), and fat-free mass (FFM, kg) were determined. Athletes were measured at the beginning of the season using one whole-body DXA scan, and the average of at least two of the measures of body weight and body height were taken [23]. For the subsample, which followed over the competition season, these measurements were repeated in the competitive phase.

### 2.6. Nutritional Intake

The athletes completed an open 7 day food record, providing information on the quantity and type of foods and fluids consumed. Total water intake was self-reported, based on the fluids and foods that were consumed. The supplements that were consumed by athletes were (1) whey protein, (2) energetic drinks, and (3) multivitamins. Each athlete documented their supplement intake as they did for their food intake, providing details on the dose and type of supplement. Then, we added the supplements to our food database and their macro- and/or micro-nutrient contents were included in the calculations of our participants’ daily intakes. Average energy, macronutrients (proteins, carbohydrates, and fat), relevant micronutrients (vitamins A, C, E, D, and B12, thiamine, riboflavin, folate, magnesium, zinc, calcium, and iron [24]) and total water intakes were estimated using Food Processor SQL (ESHA Research Inc., Salem, OR, USA). This software was supplemented with Portuguese foods and recipes. The ratio of energy intake (EI) to basal metabolic rate (BMR) was used to identify under-reporting. BMR was estimated using the Schofield equations [25]. The cut-off for under-reporting was set at 1.1 and those with a ratio smaller than that were excluded from the analyses [26]. In order to determine over-reporting, a ratio of EI to BMR of 4.0, was used as the cut-off. This correspondsed to the physical activity level (PAL) upper limit for the professional endurance athletes [27]. The reference values that were used for the micronutrients were the estimated average requirement (EAR), as defined by the Food and Nutrition Board of the Institute of Medicine (to consult the values, please see reference [11]).

### 2.7. Statistical Analysis

IBM-SPSS Statistics version 22 [28] was used for the analyses. Descriptive statistics were performed for all of the outcome measurements and were reported as mean (SD) when the data were normally distributed. The median (interquartile range) was used if the data did not present a normal distribution. The normality of the outcome variables was analyzed using the Kolmogorov–Smirnov test. A paired sample t-test was performed so as to compare the two assessment periods when the data were normally distributed, or the alternative non-parametric Wilcoxon test was performed if the data did not present a normal distribution. The percentage of inadequacy for micronutrients was calculated by the proportion of individuals whose intake was below the RDA that was adequate for their respective sex and age (age groups were defined according to the Food and Nutrition Board of the Institute of Medicine [11]). Chi-square tests were performed so as to compare the prevalence between independent groups, and Cochran’s Q-tests were performed so as to compare the prevalence between the paired samples. The association between the changes in the macro-nutrients and the related energy intake with changes in body weight and body composition were tested using Spearman’ coefficient of correlation, as at least one of the variables was not normally distributed.

## 3. Results

### 3.1. Characteristics

From the initial 276 athletes, 33 were eliminated as a result of under-reporting and 1 because of incomplete information, leaving a final sample of 242 participants. After the under-reporting, 165 athletes were evaluated only in the preparatory phase (63% males, 67.0 (15.3) kg, 175 (11) cm) and 77 (*n* = 47 for males and *n* = 30 for females) were evaluated in both of the phases (Table 1).

The whole sample in the preparatory phase represented several sports, namely: basketball (*n* = 29), judo (*n* = 24), rowing (*n* = 3), handball (*n* = 10), volleyball (*n* = 26), swimming (*n* = 92), karate (*n* = 1), triathlon (*n* = 23), tennis (*n* = 18), athletics (*n* = 4), sailing (*n* = 6), shooting (*n* = 2), fencing (*n* = 2), and horseball (*n* = 2), while the follow-up sample (athletes who were evaluated in the preparatory and competitive phase) represented basketball (*n* = 13), handball (*n* = 10), volleyball (*n* = 15), swimming (*n* = 35), and triathlon (*n* = 4).

During the preparatory phase, weight varied between 48.5–81.9 kg for females and 57.4–108.9 kg for males. The percentage of body fat and fat-free mass in the preparatory phase ranged from 17–33% and from 66–82%, respectively, for females, and from 8–26% and 73–91%, respectively, for males. In the competitive phase, the values varied between 19–33% and 67–80% for fat and fat-free mass, respectively, in females, and between 9–26% and 73–89% for body fat and fat-free mass, respectively, in males. Bone mineral content varied from 1.61–3.29 kg for females and from 1.77–4.75 kg for males, at the preparatory phase. At the competitive phase, the values were between 1.82–3.79 and 1.93–4.60 kg for females and males, respectively. Between phases, the males increased their weight, height, BMI, BMC, and FFM, while the females only increased their height.

### 3.2. Energy, Macronutrients, Micronutrients, and Fluids Intake

In the preparatory phase, 9% of females and 3% of males were below the recommendations for protein (<1.2 g/kg/d), while 16% of females and 33% of males were above (>2.1 g/kg/d). For carbohydrates and fats, 45% of females and 50% of males, and 27% of females and 18% of males, respectively, reported an intake below the minimum recommended amounts (carbohydrates <5 g/kg/d and fats <20% of total energy intake). In the competitive phase, 22% of females and 2% of males reported a protein intake below the recommendations, and 16% of females and 37% of males were above. Two-thirds (66%) and 41% of females and males, respectively, reported a carbohydrate intake below the minimum recommended amount. For fats, 25% of females and 18% of males had an intake lower than the lipid recommendations.

For males, the reported energy intake increased by 179 (−67 to 407) kcal/d (*p* = 0.003) from the preparatory phase to the competitive phase, but no difference was found for female athletes (−67 (−493 to 140) kcal/d, *p* = 0.213). The females showed a similar pattern for macro-nutrient intake between the phases, while the males reported a raise in carbohydrates—0.4 (−0.2 to 0.8) g/kg/d; *p* = 0.042—and fat—6 (−2 to 16) g/d; *p* = 0.018)—ingestion during the competitive phase, relative to the preparatory phase (Table 2).

With respect to the total water intake, 62% of the athletes in the preparatory phase and 73% of the athletes at the competitive phase reported an ingestion (from beverages and food) below the recommendations. According to sex, 69% of females and 62% of males had a water intake lower than the recommendations [8]. Females reported a decrease in water intake over the season, −356 (−712 to 78) g/d; *p* = 0.028) (Table 2).

Between the phases, females reported a decrease in the intake of vitamin A; −260 (−911 to 142) μg/d, *p* = 0.035; vitamin D, −0.7 (−1.6 to 0.0) μg/d, *p* = 0.020; and calcium, −132 (−284 to 59) mg/d, *p* = 0.021. Whereas the males reported an increase of vitamin E, 1.0 (−0.4 to 2.2) mg/d, *p* = 0.042; calcium 175 (−112 to 322) mg/d, *p* = 0.017; and magnesium, 33 (−28 to 112) mg/d, *p* = 0.024 (Table 2). Inadequate intakes of calcium, magnesium, folate, zinc, and iron were observed in more than half of the female athletes, with intakes below the recommendations in both of the phases (except for iron, which was only for the preparatory phase). More than half of male athletes had reported an intake lower than the recommendations for magnesium and folate [5]. Vitamin B12 and iron were the micro-nutrients with a lower percentage of mismatch for males, but not for females, who were below the recommended values. In the preparatory phase, females reported a higher percentage of mismatch for vitamin B12, calcium, iron, selenium, and zinc, compared with males (*p* < 0.05). In the competitive phase, a higher percentage of mismatch were reported for thiamin, vitamin B12, calcium, and iron, for females (*p* < 0.05). No differences were found between the prevalence of the micro-nutrient inadequacy from the preparatory to the competitive phase, for the macro- and micro-nutrients (Table 3).

The correlation between carbohydrate intake and protein intake (adjusted to energy intake) was −0.294 (*p* = 0.010) for the total sample (female + male). When we divided by the sexes, the value was −0.419 (*p* = 0.003) for males and −0.079 (*p* = 0.680) for females. Further analysis (see Table 4) pointed out that no association was observed between the changes in the macro-nutrients and the related energy intake, with changes in body composition (fat mass and fat-free mass) (*p* >0.05).

## 4. Discussion

This was the first study that provided information about the nutritional intakes of a large sample of Portuguese elite athletes from several sports, in the beginning of the season. Despite the individual variability for energy, macro-, and micro-nutrients, in general, the athletes reported an intake below the recommendations. In addition, a subsample was followed over the season and the main findings indicated that the males reported an increase in macro- and micro-nutrient intake, while no changes were observed in females.

Despite the fact that carbohydrates were essential for athletes so as to maintain glycaemia and to restore glycogen reserves [5], 48% in the preparatory phase and 51% in the competitive phase reported an intake below the minimum recommended value (<5 g/kg/d). Diets with severe carbohydrate restriction were popular among athletes, particularly amongst females [9], as they were usually more concerned about body size and shape than males [29]. An optimum supply of carbohydrates was one of the most important dietary requirements for athletes [15] and a restriction of that macro-nutrient has been shown to be detrimental [5], affecting performance because of the depletion of glycogen stores and/or hypoglycemia [9]. Females were usually more concerned about their body size and shape than males [30]. Even if a female athlete did not participate in a weight-related sport, the aesthetical appearance was important and carbohydrates were the macro-nutrient that was often related to weight gain, eventually being restricted from their diet [10].

The lower intake of protein was atypical, and mostly reported by females, especially in the competitive phase. Higher protein intakes than what were recommended were reported, similar to other studies [13,15]. In the preparatory phase, 34% of males and 13% of females reported a high consumption. In the competitive phase, the percentages were similar. An insufficient protein intake seemed to be mainly reported by females (17% at both phases), while for males this was uncommon. Other studies reported that athletes tended to have a protein intake that was higher than the recommendations [13,15,31]. It was common for an athlete to increase their protein to maintain skeletal muscle mass and if weight loss was needed, this macro-nutrient may have increased their satiety [32], having a special interest for athletes. However, that increase would have led to a decrease of another macro-nutrient intake, typically carbohydrates [33], and this tendency was shown in this study, specifically in the female group, and also in other studies that were mentioned above [13,15]. Nevertheless, in our female athletes, the reduction that was observed in the carbohydrate intake over the season only represented 10% of their total carbohydrates that were ingested at the preparatory phase.

Regardless of the fact that most of the athletes reported a fat intake according to their needs, 23% of females and 17% of males reported a poor consumption of fats in the preparatory phase, with a similar prevalence in the competitive phase. The consumption of ≤20% of energy from fats did not benefit performance [5]. Thus, fat-soluble vitamins and essential fatty acids were essential to an athlete’s diet [7]. Thus, the fat-soluble vitamins and essential fatty acids were essential to an athlete’s diet [2]. The IAN-AF (Inquérito Alimentar Nacional e de Atividade Física) study 2015–2016 showed that 16% and 15% of the male and female Portuguese population, respectively, reported a fat intake that was lower than the recommendations. The values of this study for polyunsaturated fats were below the recommendations, 5.4 (4.5–6.5)% of the total energy intake, although the median value for the total fat that was ingestion was 32.3 (28.8–35.9)% of the total energy intake [34]. This study also showed that the sample reported a lower intake of fish and/or seeds/nuts (sources of essential fatty acids). Therefore, the lower intake of essential fatty acids that was observed in our Portuguese athletic sample may have been the result of inadequate food choices, especially in the athletes whose total fat ingestion was below 20% of their total energy intake.

Being the macronutrient with higher energy density (9 kcal/g), reducing fat intake may be a simple way to reduce energy intake instead of decreasing another macronutrient. Furthermore, lipids are a macronutrient that does not report explicit physiological responses to performance, unlike carbohydrates and proteins [35].

Athletes reported an inadequate intake of several micro-nutrients, specifically vitamins D and E, magnesium, folate, calcium, and zinc. Females reported large inadequacy for iron and several athletes did not have an adequate intake of vitamin A, B1, B2, and B6.

Several studies showed the importance of vitamin D [36], calcium [30], and magnesium [37] for bone metabolism. Since athletes had a higher prevalence of stress fractures compared with non-athletes [38], an optimal intake of these three micronutrients was essential in order to maintain and protect their bone health. Vitamin E, being an antioxidant, had an important role in protecting the membranes from oxidative damage [39]. Females also reported a huge percentage of inadequacy for iron, thiamin, and riboflavin, and tended to make a more severe energetic restriction in order to lose weight and/or improve performance [9], being more prone to micro-nutrients deficiency [5]. In our study, females showed a higher percentage of intake below the recommendations, for both micro- and macro-nutrients. Iron was an essential nutrient for optimal oxygen delivery to the body’s tissues and for facilitating oxygen diffusion to the mitochondria, of which a deficiency might have cause fatigue, altered resistance to infection, and muscle and hormone dysfunction [40]. Similar to our results, a study made in Brazil also showed that the micro-nutrient inadequacy was higher in females when compared with males [41].

However, both genders reported a huge percentage of mismatch for micro-nutrients, and their intake needed to be optimized. Most of the vitamins and minerals participated in processes that were related to muscle contractions and energy expenditure, so it was important that the athlete presened a well-balanced diet, which covered the needs for all of the micro-nutrients in healthy humans [5]. There were few European studies that were available to compare our results with, but Wardenaar et al. (2017) showed that their Dutch elite and sub-elite athletes reported a lower intake of some micro-nutrients, such iron, vitamin D, vitamin B1 and B2, and vitamin A [10]. De Sousa et al. (2008) showed that athletes reported lower intakes than recommended for vitamin B1, vitamin E, folate, and magnesium [41]. Our results extended these findings. In addition, De Sousa et al. (2008) emphasized the importance of making good food choices for an athlete, in order to meet all of the micro-nutrient recommendations, and not just for improving performance, but also for overall health. 

The water intake was also far from the recommendations [8]. An inadequate water intake could have led to dehydration and hyponatremia [7]. A weight loss up to 4% did not show negative effects on performance [42], but athletes should be careful about their water consumption, by ensuring that they began the training euhydrated and ingesting adequate fluids during exercise so as to replace sweat losses.

We could not be certain about the influence of an inadequate nutritional intake on the athletes’ performance. We did not have performance variables to compare with the nutritional intake, however, we could speculate because of the actual literature that showed the influence of a correct nutritional intake on the performance [1]. It would have been interesting to do further research regarding this topic.

It was know that the individual’s nutritional habits (especially adolescents) were frequently inadequate, and tended to have a lower intake of dairy products, milk, vegetables, and fruits, and a large consumption of high energy-density foods, like fast food, snacks, and soft drinks, which were normally high in sugar and poor in micro-nutrients [19]. The majority of vitamins and minerals were involved in processes that were related to muscle contractions and energy expenditure, so it was important that athletes had a well-balanced diet, covering all of micro-nutrient requirements [9].

A few limitations of this study needed to be addressed. Firstly, the athletes that were followed over the season did not represent the nutritional intake that was observed by all of the sports that were assessed at the preparatory phase, limiting the generalization of these longitudinal results to sports other than basketball, handball, volleyball, swimming, and triathlon. In addition, it was not known if these two measurements (preparatory and competitive phase) represented what happened during the entire time. Although 7 day food records were considered a valid method to evaluate the nutritional intake in athletes [43], these results should have been evaluated with caution as (i) recording intakes might have caused a decrease in consumption or even an exclusion of certain types of food, to show a faultless diary [9]; and (ii) the portion sizes that were described might have been inaccurate, especially by those who did not weigh food and/or did not know common household measures, which might have led to an underestimation of the true intakes [44,45]. Additionally, this method did not allow us to understand the possible effect of seasonality on athletes’ nutritional intake. In this regard, even among the athletes who were assessed at the beginning of the season, it was not possible to guarantee that all would have been assessed within the same period. For those who were evaluated in both phases, the food choices in the preparatory phase might have been different from the competitive phase as a result of seasonal changes in the food products that were available. Increased accuracy cold be achieved with a longer duration of the food intake recording. However, this must have been balanced with the participant burden and fatigue, which could have reduced compliance and accurate reporting. Other dietary assessment tools could have been used to assess the long-term intakes, but those were usually less precise (for example, a food frequency questionnaire). In this case, 7 d of recordings were considered quite adequate so as to capture the intake estimates of the macro-nutrients and most of the micro-nutrients [45]. Lastly, the statistical power to detect the nutritional intake inadequacy was not determined, as this sample was a part of a larger study that was conducted to test if body composition was associated with performance in physical tests over a season. Thus, the ability to detect some differences in nutritional intake might have been compromised.

## 5. Conclusions

In conclusion, the male and female athletes from several sports reported a macro- and micro-nutrient intake below the recommendations at the preparatory phase. Over the season, males increased their lipids, carbohydrates, and overall energy intake by 6%, 9%, and 6%, respectively, whereas no differences were found for females. Nevertheless, these gender differences may not be relevant, given the large individual variability that was observed. These findings revealed that attention should be given to the athletes’ nutritional intake, especially in females, so as to avoid compromising health and performance.

## Author Contributors

A.M.S. and L.B.S. contributed to the study design. D.A.S., C.N.M., J.P.M., C.S.M., C.P.M., and L.B.S. contributed to conducting the research. C.L.N was the principal author and carried out the data analyses and wrote the manuscript. C.L.N., C.N.M., D.A.S., M.S., C.P.M., C.S.M., J.P.M., M.P.S. and P.M.R. contributed to data analyses. M.S., A.M.S., and M.P.S. contributed to the writing of the manuscript. All of the authors read and approved the final version of the manuscript.

## Figures and Tables

**Table 1:** Characteristics of athletes at the preparatory and competitive phase of the season, according to sex.

	Male					Female				
	Whole Sample ^†^ (*n* = 153)	Follow-Up ^‡^ (*n* = 47)				Whole Sample ^†^ (*n* = 89)	Follow-Up ^‡^ (*n* = 30)			
	Preparatory Phase	Preparatory Phase	Competitive Phase	Differences ^§^	*p* Value	Preparatory Phase	Preparatory Phase	Competitive Phase	Differences ^§^	*p* Value
Age, median (IQR), year	20 (18; 25)	18 (16; 20)	20 (18; 23)	NA		17 (16; 20)	16 (15; 19)	18 (16; 24)	NA	
Weight, kg	72.4 (10.5)	78.5 (11.1)	79.3 (10.6)	1.2 (2.4) *	0.002	58.5 (8.5)	62.9 (9.2)	63.7 (9.1)	0.5 (1.8)	0.134
Height, cm	179.5 (9.4)	183.3 (10.5)	183.8 (9.6)	0.5 (0.8) *	<0.001	166.8 (7.8)	167.2 (6.9)	167.6 (6.8)	0.4 (0.6)*	<0.001
BMI, kg/m^2^	22.6 (2.1)	22.6 (2.3)	22.8 (2.2)	0.23 (0.69) *	0.026	21.2 (2.1)	21.7 (2.1)	21.8 (2.0)	0.69 (4.29)	0.385
BMC, kg ^||^	3.12 (0.65)	3.15 (0.68)	3.19 (0.69)	0.04 (0.10) *	0.019	2.34 (0.41)	2.38 (0.35)	2.38 (0.36)	0.00 (0.76)	0.831
FFM, kg ^||^	65.0 (8.5)	66.5 (9.0)	67.4 (9.0)	1.1 (1.5) *	<0.001	46.0 (5.9)	46.6 (6.6)	47.7 (6.3)	0.5 (1.8)	0.121
FM, median (IQR), kg ^||^	9.3 (7.8; 11.7)	10.3 (8.5; 13.2)	10.8 (9.4; 12.8)	0.22 (−1.14; 0.73)	0.783	14.8 (12.9; 17.9)	15.0 (13.2; 17.3)	14.3 (12.6; 17.7)	−0.75 (−1.07; 0.22)	0.053
FM, median (IQR), % ^||^	12.6 (11.1; 15.1)	14.0 (12.5; 16.7)	14.1 (11.9; 15.4)	0.30 (−1.02; 0.48)	0.849	24.4 (22.5; 27.4)	25.5 (22.6; 27.5)	24.1 (21.2; 26.3)	−0.79 (−1.75; −0.30)	0.128

Data presented as mean (SD) unless otherwise indicated. Abbreviations: NA—non-applicable; BM—body mass index; BMC—bone mineral content; FM kg—fat mass (kilograms); FM %—fat mass (percentage); FFM—fat-free mass; IQR—interquartile range. ^†^ Whole sample including all of the athletes with valid data for the preparatory phase, including those that were followed-up over the season. ^‡^ Data for athletes that were assessed both at the preparatory and competitive phases. ^§^ Mean difference between the competitive and preparatory phases (CP–PP) (follow-up). Data presented as median (interquartile). ^||^ Performed in a subsample. For age and for the absolute and relative values of fat mass median and interquartile, are presented instead of mean (SD). * Significantly different between preparatory and competitive phases, *p* < 0.05.

**Table 2:** Energy, macro-nutrients, and micro-nutrients intake at the preparatory and competitive phase of the season, by sex.

	Male					Female				
	Whole Sample ^†^ (*n* = 153)	Follow-Up ^‡^ (*n* = 47)				Whole Sample ^†^ (*n* = 89)	Follow-Up ^‡^ (*n* = 30)			
	Preparatory Phase	Preparatory Phase	Competitive Phase	Differences ^§^	*p* Value	Preparatory Phase	Preparatory Phase	Competitive Phase	Differences ^§^	*p* Value
Energy, kcal/d	2961 (2585–3559)	2976 (2628–3495)	2146 (2886–3585)	179 (−67; 407) *	0.003	2373 (2059–2673)	2425 (2134–2761)	2349 (1941–2669)	−67 (−493; 140)	0.213
Proteins, g/kg/d	1.9 (1.5–2.2)	1.8 (1.5–2.3)	2.0 (1.6–2.2)	0.1 (−0.2; 0.3)	0.257	1.8 (1.5–1.8)	1.8 (1.5–2.0)	1.6 (1.4–1.9)	−0.1 (−0.3; 0.2)	0.289
CHO, g/kg/d	5.0 (3.9–6.8)	4.6 (3.8–6.1)	5.2 (4.1–6.0)	0.4 (−0.2; 0.8) *	0.042	5.1 (4.4–6.2)	5.1 (4.4–6.6)	4.8 (3.6–5.1)	−0.5 (−1.1; 0.5)	0.058
Lipids, g/d	102 (86–120)	106 (93–119)	116 (100–127)	6 (−2; 16) *	0.018	79 (66–95)	86 (71–101)	78 (65–101)	−6 (−25; 18)	0.323
Water, g/d	2141 (1575–3119)	2029 (1572–2847)	2300 (1605–2833)	−112 (−414; 401)	0.791	1846 (808–3807)	1755 (1432–2179)	1423 (1064–1989)	−356 (−712; 78) *	0.028
Vitamin A, μg/d	1315 (726–2066)	1232 (726–1738)	1145 (706–1712)	−106 (−520; 329)	0.330	1221 (731–2008)	1316 (667–1979)	835 (455–1355)	−260 (−911; 142) *	0.035
Thiamin, mg/d	1.6 (1.2–2.1)	1.5 (0.9–1.8)	1.4 (1.1–1.8)	0.0 (−0.4; 0.5)	0.772	1.1 (0.9–1.5)	1.0 (0.9–1.3)	1.0 (0.8–1.2)	0.0 (−0.3; 0.3)	0.447
Riboflavin, mg/d	1.6 (1.1–2.4) ^#^	1.3 (0.8–1.9)	1.3 (0.9–1.7)	0.1 (−0.3; 0.5)	0.874	1.0 (0.8–1.3)	1.0 (0.7–1.3)	1.0 (0.7–1.1)	0.0 (−0.3; 0.2)	0.767
Niacin, mg/d	25.7 (10.0–58.7)	29.4 (19.6–49.9)	26.4 (10.2–52.1)	−1.4 (−30.4; 28.1)	0.427	25.8 (9.0–52.8)	22.9 (12.5–58.7)	27.9 (17.8–51.7)	6.0 (−27.3; 29.9)	0.096
Pyridoxine, mg/d	1.9 (1.5–2.7)	1.7 (1.1–2.3)	1.5 (1.1–2.0)	−0.2 (−0.6; 0.4)	0.310	1.2 (2.8–6.8)	1.2 (1.0–1.6)	1.2 (0.9–1.6)	0.0 (−0.4; 0.4)	0.943
Vitamin B12, μg/d	6.8 (4.2–10.2)	6.0 (3.8–9.4)	5.1 (3.4–8.9)	0.2 (−3.3; 2.2)	0.649	4.0 (0.2–24.0)	3.2 (2.5–4.8)	3.3 (2.4–5.6)	0.0 (−2.1; 2.8)	0.600
Vitamin C, mg/d	103 (71–159)	92 (62–139)	105 (63–141)	6.9 (−26.1; 60.0)	0.150	90 (65–137)	77 (59–123)	90 (43–155)	0.3 (−23.0; 71.6)	0.262
Vitamin D, μg/d	3.1 (1.8–4.9)	2.6 (1.5–3.7)	2.9 (1.7–4.7)	0.2 (−1.1; 2.2)	0.085	2.5 (1.7–3.9)	2.5 (1.7–3.4)	1.8 (1.1–2.7)	−0.7 (−1.6; 0.0) *	0.020
Vitamin E, mg/d	4.0 (2.6–6.2)	3.4 (2.5–5.2)	4.7 (3.4–5.9)	1.0 (−0.4; 2.2) *	0.042	3.6 (2.7–5.1)	3.9 (2.8–5.2)	3.7 (2.5–5.5)	0.2 (−1.3; 2.1)	0.443
Folate, μg/d	354 (273–477)	333 (275–405)	356 (286–459)	34 (−49; 96)	0.156	290 (234–414)	284 (237–426)	268 (197–348)	−15 (−90; 30)	0.185
Calcium, mg/d	1085 (857–1379)	1075 (827–1269)	1109 (932–1528)	175 (−112; 322) *	0.017	864 (576–1007)	890 (578–994)	710 (499–864)	−132 (−284; 59) *	0.021
Iron, mg/d	16.4 (13.8–21.5)	15.6 (12.6–19.9)	17.1 (13.2–21.8)	1.4 (−3.1; 4.9)	0.240	12.3 (10.0–16.0)	11.9 (9.4–17.2)	11.5 (9.7–13.5)	−0.1 (−3.7; 2.6)	0.558
Magnesium, mg/d	304 (253–385) ^#^	288 (243–329)	319 (272–375)	33 (−28; 112) *	0.024	243 (206–296)	239 (210–308)	239 (189–279)	−15 (−52; 50)	0.629
Zinc, mg/d	10.8 (9.4–14.4)	10.4 (8.6–13.3)	11.5 (8.8–15.6)	0.5 (−1.4; 4.3)	0.137	7.5 (6.5–9.3)	7.3 (6.3–9.5)	8.0 (6.3–9.6)	0.5 (−0.9; 1.9)	0.202

Data are presented as median (interquartile range). Abbreviations: CHO—carbohydrates. ^†^ Whole sample included all of the athletes with valid data for the preparatory phase, including those that were followed-up over the season. ^‡^ Data for athletes that were assessed both at the preparatory and competitive phases. ^§^ Difference between competitive and preparatory phases (for the follow-up sample) according to sex. * Significantly different between preparatory and competitive phases. *p* < 0.05; ^#^ Significantly different between the whole sample and the followed-up sample (subsample), *p* < 0.05.

**Table 3:** Percentage of athletes who reported an intake that was lower than the recommendations for each macro- and micro-nutrient † at the preparatory and competitive phase of the season, according to sex.

	Total			Male			Reference Values ^##^	Female			Reference Values ^##^
	Whole Sample ^‡^	Follow-Up ^§^		Whole Sample ^‡^	Follow-Up ^§^			Whole Sample ^‡^	Follow-Up ^§^		
	PP	PP	CP	PP	PP	CP		PP	PP	CP	
Proteins, g/kg/d	5.8	10.4	7.8	3.9	6.4	2.1	1.2–2.1	9.0	16.7	16.7	1.2–2.1
Carbohydrates, g/kg/d	47.9	51.9	49.4	49.7	55.3	40.4	≥5	44.9	46.7	63.3	≥5
Lipids, %/d	21.5	19.5	20.8	18.3	17.0	17.0	20–35	27.0	23.3	26.7	20–35
Vitamin A, μg/d	29.8	33.8	41.6	34.0	38.3	38.8	1000	22.5	26.7	46.7	800
Thiamin, mg/d	28.5 ^#^	44.2	36.4	23.5	27.7	26.5	1.5	37.1	46.7	50.0 *	1.1
Riboflavin, mg/d	31.0 ^#^	44.2	44.2	26.8	40.4	38.8	1.7	38.2	46.7	50.0	1.3
Niacin, mg/d	4.0	1.3	3.9	4.0	0.0	6.4	19	4.1	3.0	0.0	15
Pyridoxine, mg/d	29.3	36.8	39.0	20.9	34.0	36.2	2.0	43.8	33.3	43.3	1.6
Vitamin B12, μg/d	7.0	11.7	10.4	2.6	4.3	4.1	2.2	14.6	23.3 *	20.0 *	2.2
Vitamin C, mg/d	25.6	31.2	32.5	25.5	34.0	34.7	60	25.8	33.3	30.0	60
Vitamin D, μg/d	99.2	100.0	100.0	98.7	100.0	100.0	<25 y: 10 ≥25 y: 5	100.0	100.0	100.0	<25 y: 10 ≥25 y: 5
Vitamin E, mg/d	97.1	97.4	100.0	96.1	100.0	100.0	10	98.9	100.0	100.0	8
Folate, μg/d	69.4	72.7	68.8	66.7	61.7	63.3	200	74.2	73.3	80.0	180
Calcium, mg/d	66.1	74.0	66.2	52.3	51.1	53.1	<25 y: 1200 ≥25 y: 800	89.9	96.7 *	90.0 *	<25 y: 1200 ≥25 y: 800
Iron, mg/d	28.5	32.5	33.8	9.2	6.4	6.1	10	61.8	60.0 *	76.7 *	15
Magnesium, mg/d	82.6 ^#^	92.2	85.7	81.0	85.1	85.7	350	85.4	90.0	86.7	280
Zinc, mg/d	56.6	59.7	49.4	48.4	42.6	42.9	15	70.8	66.7	60.0	12

Data presented as %. Abbreviations: PP—preparatory phase; CP—competitive phase. ^†^ Values represent the number of athletes (as percentage) that are below the recommendations, ranging between 0% and 100%. ^‡^ Whole Sample included all of the athletes with valid data for the preparatory phase, including those that were followed-up over the season. ^§^ Data for the athletes that were assessed both at the preparatory and competitive phases. * Significant differences between sexes at the same phase (*p* < 0.05). ^#^ Significantly different between the whole sample and the follow-up sample (subsample), *p* < 0.05. ^##^ The reference values only represent the range of our sample for males/females.

**Table 4:** Association between changes in macro-nutrients and related energy intake with changes in body composition (Spearman’ correlation).

		Energy (kcal/d)	*p*	CHO (g/kg/d)	*p*	Protein (g/kg/d)	*p*	Lipids (%/d)	*p*
		r		r		r		r	
**Male**	%BF	0.033	0.821	−0.024	0.874	−0.203	0.172	0.097	0.097
	%FFM	−0.034	0.826	−0.077	0.605	−0.146	0.327	−0.130	0.385
**Female**	%BF	−0.128	0.501	−0.192	0.311	−0.108	0.568	−0.088	0.643
	%FFM	−0.040	0.833	−0.063	0.740	−0.214	0.214	−0.046	0.811

Abbreviations: %BF—difference of percentage of body fat between competitive and preparatory phase (CP–PP). %FFM—difference of percentage of fat-free mass between competitive and preparatory phase (CP–PP). CHO—carbohydrates; r—correlation coefficient.
